# circATP2A2 promotes osteosarcoma progression by upregulating MYH9

**DOI:** 10.1515/med-2021-0370

**Published:** 2021-11-22

**Authors:** Xin Cao, Xianfeng Meng, Peng Fu, Lin Wu, Zhen Yang, Huijin Chen

**Affiliations:** Department of Trauma and Orthopaedics, Shengli Oilfield Central Hospital, Dongying, Shandong, China; Department of Clinical Laboratory, Shengli Oilfield Central Hospital, No. 31, Jinan Road, Dongying, 257000, Shandong, China

**Keywords:** OS, circATP2A2, miR-335-5p, MYH9

## Abstract

Osteosarcoma (OS) is a highly metastatic primary malignant tumor. CircRNA hsa_circ_0028173 (circATP2A2) has been uncovered to be related to the advancement of OS. However, the biological role of circATP2A2 in OS has not been validated. circATP2A2 and MYH9 were upregulated while miR-335-5p was downregulated in OS. OS patients with high circATP2A2 expression displayed a shorter overall survival and the area under curve of circATP2A2 was 0.77, manifesting that circATP2A2 might be a diagnostic and prognostic biomarker. circATP2A2 silencing repressed OS cell proliferation and glycolysis *in vivo* and constrained OS cell proliferation, glycolysis, migration, and invasion *in vitro*. circATP2A2 regulated MYH9 expression through sponging miR-335-5p. MiR-335-5p inhibitor reversed the repressive effect of circATP2A2 knockdown on OS cell malignancy and glycolysis. MYH9 overexpression overturned miR-335-5p upregulation-mediated OS cell malignancy and glycolysis. circATP2A2 accelerated OS cell malignancy and glycolysis through upregulating MYH9 via sponging miR-335-5p, offering a promising target for OS treatment.

## Graphical abstract

**Figure:**
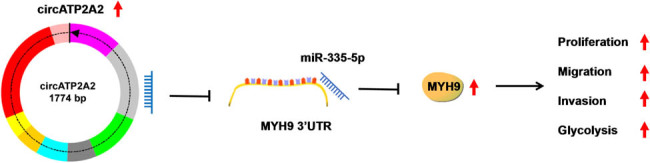

## 1 Introduction

Osteosarcoma (OS) is a common primary malignant tumor that occurs mainly in children and adolescents near the shoulder joint and the long bone metaphysis [1]. Also, diffusion to the lungs is the leading cause of OS death [2]. With the advancement of medical technologies, the long-overall survival rates of OS patients have enhanced conspicuously to 65–70% [3]. So far, the exact molecular mechanism of the occurrence and progression of OS is still unclear. Therefore, exploring the molecular mechanism of OS progression is crucial to finding specific targets for OS.

Changes in energy metabolism are one of the hallmarks of cancer, including OS. The metabolism of cancer cells is characterized by preferential reliance on glycolysis, which produces low adenosine triphosphate (ATP), but cancer cells can compensate for this deficiency by increasing glucose uptake [4]. Studies have shown that inhibiting malignant cells with increased glycolytic capacity may be a key anti-cancer strategy [5]. Therefore, it is very important to explore the mechanism related to glycolysis in OS.

Circular RNAs (circRNAs), which have a covalent closed-loop structure, are important elements for regulating the genome [6]. CircRNAs can function as microRNA (miR) sponges and decrease miR activity by binding to miRs [7,8]. Recent research studies have proved that circRNAs exert crucial roles in the initiation and advancement of human diseases, especially in cancer [9]. For instance, circRNA TADA2A upregulation promoted OS cell growth and metastasis by increasing CREB3 expression [10]. CircRNA FAT1 facilitated the tumorigenesis of OS by adsorbing miR-375 and increasing YAP1 expression [11]. CircRNA hsa_circ_0028173 (circATP2A2), located on chr12: 110764194-110780253, is derived from the ATPase sarcoplasmic/endoplasmic reticulum Ca^2+^ transporting 2 (ATP2A2) gene. However, the biological role of circATP2A2 in OS has not been verified.

MiRs are short noncoding RNAs (20–22 nucleotides) that regulate gene expression [12] and participate in a battery of biological processes [13]. The dysregulation of miRs is related to the occurrence of many diseases [14]. MiR-335-5p has been revealed to play a suppressive role in various tumors, such as lung adenocarcinoma [15], thyroid cancer [16], and colorectal cancer [17]. MiR-335-5p also exerted an inhibitory effect in OS [18]. However, the mechanism of the abnormal expression of miR-335-5p in OS is unclear.

Non-muscle myosin (NM IIA), a cytoplasmic myosin, was involved in several processes that require actin cytoskeletal translocation and intracellular chemical mechanical forces [19]. The myosin heavy chain 9 (MYH9) gene, which encodes an NM IIA heavy chain, is associated with human diseases [19]. MYH9 has been uncovered to be connected with tumor progression, tumor differentiation, lymph node metastasis, and poor prognosis [20]. It was reported that high MYH9 expression was associated with OS cell metastasis and invasion [21]. Nevertheless, the mechanism that regulates MYH9 in OS has not been fully explained.

Herein, we validated circATP2A2 as an oncogene in OS. Also, circATP2A2 promoted OS cell malignancy and glycolysis by regulating miR-335-5p/MYH9. Our research provided a novel mechanism for understanding the progression of OS.

## 2 Materials and methods

### 2.1 OS samples

This research was permitted by the Ethics Committee of Shengli Oilfield Central Hospital. Blood samples were from 51 OS patients and 47 healthy people. Fifty-one paired OS tissues and adjacent normal bone tissues were obtained from 51 primary OS patients who were diagnosed at Shengli Oilfield Central Hospital. All patients signed informed consent and did not receive chemotherapy or radiotherapy before surgery. The clinic-pathological information of the 51 OS patients is exhibited in Table 1.

**Table 1:** Clinic-pathological information of the 51 OS patients

Clinicopathological features	Number of cases
Age	
>20 years	19
≤20 years	32
Gender	
Male	35
Female	16
Location	
Femur/Tibia	30
Elsewhere	21
Tumor size (cm)	
>8	22
≤8	29
Enneking stage	
I	6
II	27
III	16
Tumor metastasis	
Present	18
Absent	33

### 2.2 Cell culture and transfection

OS cell lines (U2OS, HOS, and Saos-2) and the hFOB1.19 cells were obtained from Procell (Wuhan, China). These cells were cultured in McCoy’s 5A (for U2OS and Saos-2 cells) (Procell), minimum essential medium (MEM) (for HOS cells) (Procell), or Dulbecco’s modified Eagle’s medium/Ham’s F12 medium (DMEM/F12) (for hFOB1.19 cells) (Procell) supplemented with 10% fetal bovine serum (FBS) (Procell) and 1% penicillin/streptomycin (Procell). All cell lines were cultivated in an incubator with 5% CO_2_ at 37°C.

Small hairpin (shRNA) negative control (sh-NC) and circATP2A2 shRNA (sh-circATP2A2) were obtained from Genepharma (Shanghai, China). The full length of MYH9 was inserted into the pcDNA3.1 vector (Invitrogen) to construct the pcDNA-MYH9 vector (MYH9). The MiR-335-5p inhibitor (anti-miR-335-5p), miR-335-5p mimic (miR-335-5p), and their matched negative control (anti-NC and miR-NC) were purchased from Ribobio (Guangzhou, China). Cell transfection was performed using the Lipofectamine 3000 reagent (Invitrogen).

### 2.3 Real-time reverse transcription polymerase chain reaction (RT-qPCR)

The TRIzol reagent (Invitrogen) was utilized to isolate total RNA from the serum, tissues, and cells. The high-capacity complementary DNA Reverse Transcription Kits (Invitrogen) or MicroRNA Reverse Transcription Kit (Invitrogen) was used for cDNA generation. The SYBR Green PCR Master Mix (Vazyme, Nanjing, China) was utilized for qPCR analysis. Primer sequences were synthesized by Ribobio (Table 2). Expression levels of circATP2A2, miRs, or MYH9 mRNA were calculated using the 2^−ΔΔ*C*t^ method, and β-actin or U6 was deemed as an internal control.

**Table 2:** Primer sequences utilized for RT-qPCR

Genes	Primer sequences (5′-3′)
circATP2A2	Forward (F): 5′-TCCGCTACCTCATCTCGT-3′
	Reverse (R): 5′-GTTGCTACCACCACTCCC-3′
MYH9	F: 5′-ATCCTGGAGGACCAGAACTGCA-3′
	R: 5′-GGCGAGGCTCTTAGATTTCTCC-3′
miR-335-5p	F: 5′-CGCGTCAAGAGCAATAACGAA-3′
	R: 5′-GAACATGTCTGCGTATCTC-3′
miR-142-5p	F: 5′-CATAAAGTAGAAAGCACTAC-3′
	R: 5′-GAACATGTCTGCGTATCTC-3′
miR-145-5p	F: 5′-GTCCAGTTTTCCCAGGA-3′
	R: 5′-GAACATGTCTGCGTATCTC-3′
miR-338-3p	F: 5′-CGCGTCCAGCATCAGTGATT-3′
	R: 5′-GAACATGTCTGCGTATCTC-3′
miR-95-3p	F: 5′-CGCGTTCAACGGGTATTTAT-3′
	R: 5′-GAACATGTCTGCGTATCTC-3′
β-actin	F: 5′-AAATCTGGCACCACACCTTC-3′
	R: 5′-GGGGTGTTGAAGGTCTCAAA-3′
U6	F: 5′-GCTTCGGCAGCACATATACTAAAAT-3′
	R: 5′-CGCTTCACGAATTTGCGTGTCAT-3′

### 2.4 Cell proliferation analysis

The proliferation of OS cells was assessed by 3-(4,5-dimethylthiazol-2-YI)-2,5-diphenyltetrazolium bromide (MTT) and plate clone assays. For the MTT assay, the transfected OS cells (5 × 10^3^) were cultured in 96-well plates (Corning Costar, Corning, NY, USA) for 24, 48, or 72 h. After that, the MTT solution (20 μL, 5 mg/mL) (Sigma, St. Louis, MO, USA) was added to each well, followed by dissolving the formaldehyde crystals using dimethyl sulfoxide (100 μL) (Sigma). Finally, the color reaction at 490 nm was analyzed by the microplate detection system (Molecular Devices, San Jose, CA, USA).

For the plate clone assay, the transfected OS cells (1.5 × 10^2^) were cultured in 6-well plates (Corning) for 14 days, followed by fixing with paraformaldehyde and staining with 0.1% crystal violet (Sigma). The colonies were counted using a microscope (Nikon Instruments, Melville, NY, USA).

### 2.5 Flow cytometer assay

The transfected OS cells were collected and then detached with 0.025% trypsin (Invitrogen), followed by fixing with 70% ethanol (Sigma). Subsequently, the cells (1 × 10^6^) were stained with propidium iodide (PI) solution (400 μL) consisting of 4 µg/mL PI (Sigma), 0.5 mg/mL RNase A (Invitrogen), and 1% Triton X-100 (Sigma). The cellular DNA content was assessed with a FACS Verse flow cytometer (Becton Dickinson, San Jose, CA, USA) and analyzed with FlowJo software (FlowJo v7.6, LLC, Ashland, OR, USA).

### 2.6 Transwell assay

Transwell chambers (Corning) were used for migration and invasion analysis. In short, the transfected OS cells (1 × 10^5^) were resuspended in McCoy’s 5A medium without FBS and then placed in the apical chamber. The McCoy’s 5A medium containing 10% FBS (Procell) was added to the basolateral chamber. After 24 h, the migrating and invading cells were stained with 0.1% crystal violet (Sigma), followed by counting with a microscope (Nikon Instruments).

### 2.7 Metabolism analysis

The levels of glucose uptake and lactate product in OS cells were detected using the glucose uptake colorimetric assay kit (BioVision, Milpitas, CA, USA) or lactate assay kit II (BioVision) based on the manufacturer’s instructions.

### 2.8 Western blot analysis

Total protein was extracted with RIPA lysis buffer (Beyotime, Shanghai, China) and then quantified with the BCA assay kit (Pierce, Rockford, IL, USA). Sodium dodecyl sulfate-polyacrylamide gel electrophoresis (SDS-PAGE) (8–12%) was conducted for total protein segregation. Then, the segregated proteins were transferred onto the polyvinylidene difluoride (PVDF) membranes (Millipore, Billerica, MA, USA) and then blocked with Tris-buffered saline Tween buffer containing 5% skim milk. The PVDF membranes were then incubated with primary antibodies against hexokinase 2 (HK2) (#PA5-29326, 1:5,000, Invitrogen), pyruvate kinase M2 (PKM2) (#PA5-28700, 1:500, Invitrogen), MYH9 (#PA5-29673, 1:1,000, Invitrogen), and β-actin (#PA5-11570, 1:1,000, Invitrogen). Moreover, β-actin was regarded as a loading control. The membranes were then washed and incubated with goat anti-rabbit IgG (#31460, 1:10,000, Invitrogen). The blots were developed using Pierce Super Signal West Dura.

### 2.9 RNA pulldown assay

The circATP2A2 probe and oligo probe were designed and synthesized by RiboBio, followed by incubating with C-1 magnetic beads (Life Technologies, Carlsbad, CA, USA). Then, the lysates of OS cells were incubated with C-1 magnetic beads. RNA complexes bound to the C-1 magnetic beads were eluted and then analyzed via RT-qPCR.

### 2.10 Bioinformatics analysis

The candidate miRs containing the putative binding sites of circATP2A2 were analyzed using circinteractome, starBase, and GSE28423 databases. The binding sites between MYH9 and miR-335-5p were predicted by the starBase database.

### 2.11 Dual-luciferase reporter assay

The sequences of circATP2A2-wt, circATP2A2-mut, MYH9 3′UTR (untranslated region)-wt, and MYH9 3′UTR-mut were synthesized and then inserted into the psiCHECK2 vector (Promega, Madison, WI, USA), respectively. OS cells were co-transfected with miR-335-5p mimic or miR-NC and a luciferase plasmid carrying circATP2A2-wt, circATP2A2-mut, MYH9 3′UTR-wt, or MYH9 3′UTR-mut. The luciferase activities were evaluated using a dual-luciferase reporter assay kit (BioVision).

### 2.12 RIP (RNA immunoprecipitation) assay

RIP assay was conducted with the Magna RIP kit (Millipore, Bedford, MA, USA) in accordance with the manufacturer’s instructions. The lysates of OS cells were incubated with immunoprecipitation buffer containing magnetic beads conjugated to IgG antibody (Abcam, Cambridge, MA, USA) or Ago2 antibody (Abcam). The enrichment of circATP2A2 or MYH9 and miR-335-5p was assessed by RT-qPCR.

### 2.13 Immunohistochemistry (IHC)

The paraffin-embedded tissue was sectioned into 4 μm slides, followed by performing IHC in accordance with the previously described procedure [22]. The sections were incubated with anti-MYH9 (#PA5-29673, 1:500, Invitrogen) or ki67 (#27309-1-AP, 1:4,000, Invitrogen) antibodies.

### 2.14 Xenograft assay

The experiment was authorized by the Animal Ethics Committee of Shengli Oilfield Central Hospital. Ten BALB/c nude mice (4–6 weeks old) were obtained from Vital River Laboratory (Beijing, China) and divided into 2 groups (*n* = 5) by random number table. Briefly, Saos-2 cells (5 × 10^6^ cells/100 μL) with stable knockdown of circATP2A2 were injected into nude mice (*n* = 5), and sh-NC was used as a control. The tumor volume was measured with a digital caliper every 7 days and calculated with the following equation: volume = (length × width^2^)/2. After 28 days, the mice were euthanized under anesthesia to obtain the tumor tissues for subsequent analysis.

### 2.15 Statistical analysis

SPSS 18.0 (SPSS, Chicago, IL, USA) was employed for statistical analysis. Data were produced from three independent experiments and presented as mean ± standard deviation. The Kaplan–Meier method with the log-rank (Mantel–Cox) test was utilized for analysis of the overall survival rate of OS patients. Receiver operating characteristic (ROC) curves were plotted using GraphPad Prism 5 (GraphPad Software 5.0, LaJolla, CA). The Student’s *t*-test or one-way analysis of variance was used to compare the differences between two groups and more than two groups. When *P*＜0.05, the data were deemed statistically significant.

## 3 Results

### 3.1 circATP2A2 might be a diagnostic and prognostic biomarker for OS patients

**Figure 1:**
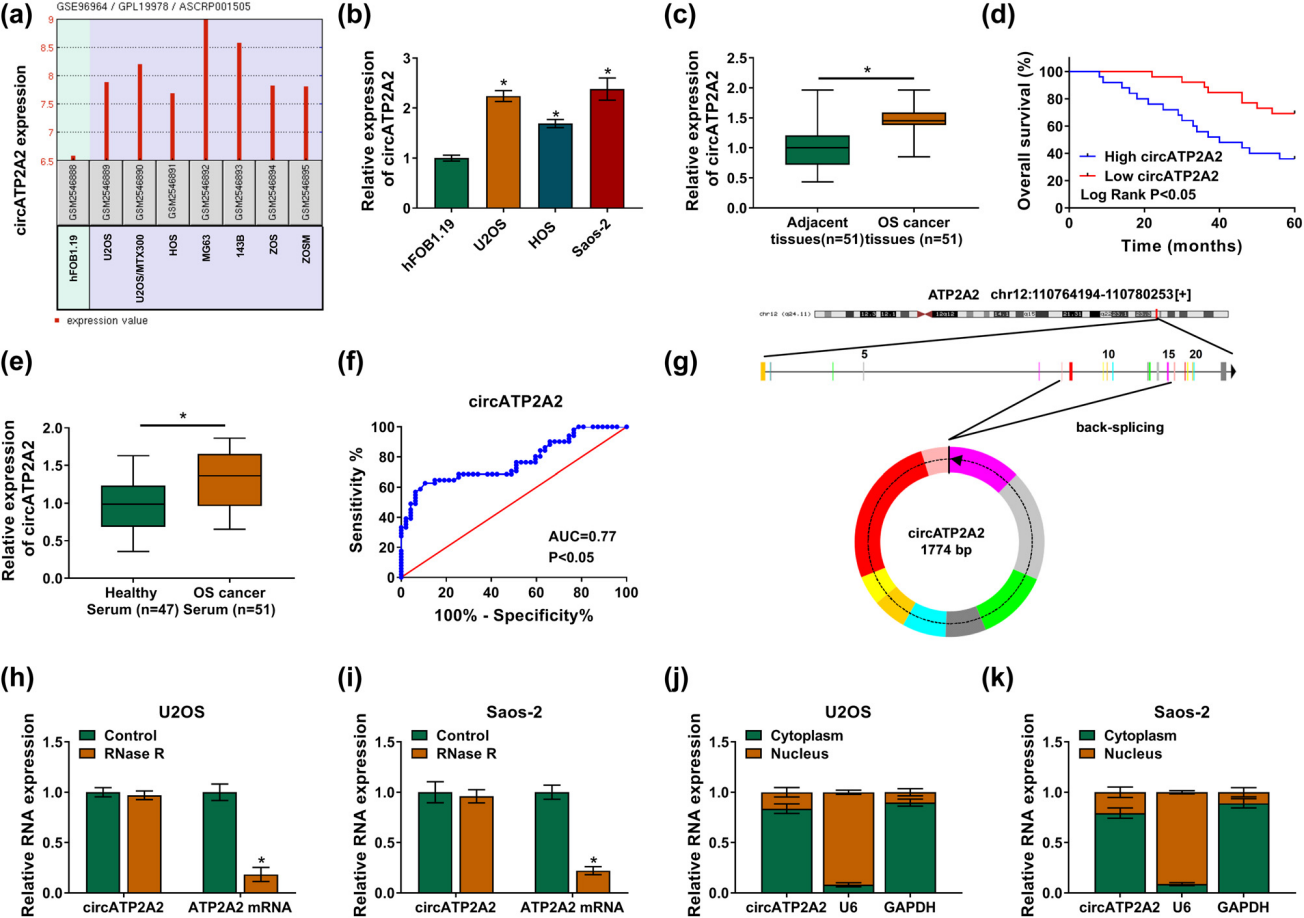
circATP2A2 might be a diagnostic and prognostic biomarker for OS patients. (a) The expression pattern of circATP2A2 in the GSE96964 dataset. (b and c) The expression of circATP2A2 in OS cells and tissues was detected by RT-qPCR. (d) Kaplan–Meier analysis revealed the overall survival rate of OS patients with high or low circATP2A2 expression. The median value of circATP2A2 (the median value was 1.452) in OS tissues based on RT-qPCR was used as the cutoff to define “low” or “high” circATP2A2 expression. (e) The expression of circATP2A2 in the serum of healthy people (47) and OS patients was assessed by RT-qPCR (51). (f) The diagnostic value of circATP2A2 in serum was analyzed by the ROC curve. (g) The schematic diagram shows the formation of circATP2A2. (h and i) After RNase R treatment, the level of circATP2A2 in OS cells was analyzed by RT-qPCR. (j and k) The abundance of circATP2A2 in the cytoplasm and nucleus of OS cells was assessed by RT-qPCR. *P < 0.05.

By analyzing the GSE96964 dataset downloaded from the GEO database (https://www.ncbi.nlm.nih.gov/geo/query/acc.cgi?acc=GSE96964), we found that circATP2A2 was a differentially expressed circRNA in OS (Figure 1a). To survey the biological role of circATP2A2 in OS, we detected circATP2A2 expression in OS. Expression of circATP2A2 was apparently increased in OS cells (U2OS, HOS, and Saos-2) and tissues in contrast to their matched control groups (Figure 1b and c). Also, Kaplan–Meier curves and log-rank (Mantel–Cox) test were used for survival analysis, and OS patients with low circATP2A2 expression displayed a higher overall survival (Figure 1d). In addition, circATP2A2 was drastically upregulated in the serum of OS patients (Figure 1e). The ROC curve analysis exhibited that the area under curve (AUC) of circATP2A2 was 0.77, manifesting that circATP2A2 might be a clinical diagnostic biomarker for OS (Figure 1f). circATP2A2 is derived from the reverse splicing of the ATP2A2 gene and forms a 1,774 bp circular transcript (Figure 1g). Moreover, circATP2A2 was resistant to RNase R treatment (Figure 1h and i). Additionally, circATP2A2 was more distributed in the cytoplasm of OS cells (Figure 1j and k). These results indicated that circATP2A2 might be related to OS progression.

### 3.2 circATP2A2 facilitated OS cell malignancy and glycolysis

**Figure 2:**
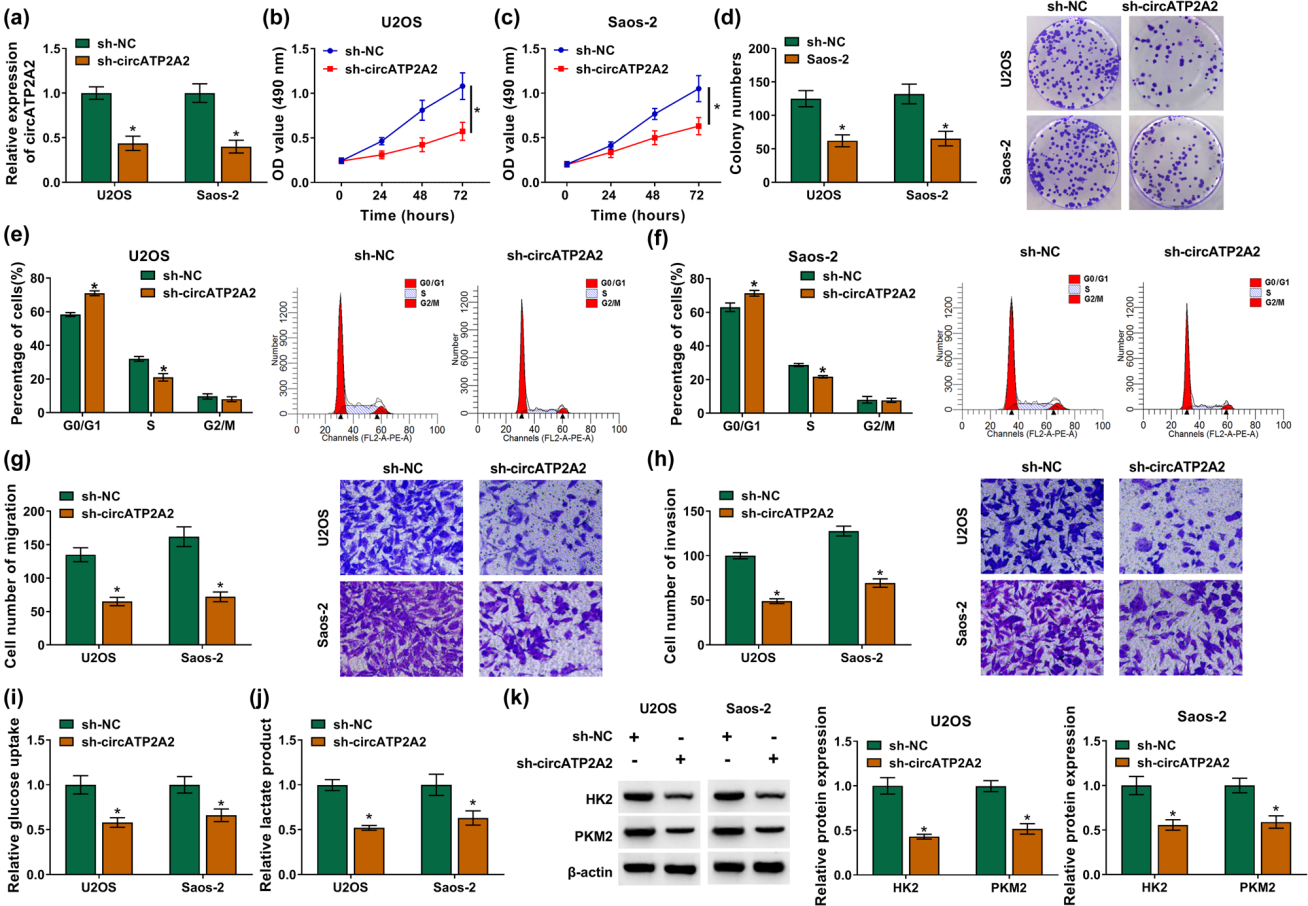
circATP2A2 promoted OS cell malignancy and glycolysis. (a–k) OS cells were transfected with sh-NC or sh-circATP2A2. (a) The interference efficiency of sh-circATP2A2 in OS cells was verified by RT-qPCR. (b–h) The proliferation, cell cycle progression, migration, and invasion of OS cells were evaluated by MTT, plate clone, flow cytometry, and transwell assays, respectively. (i and j) Assessment of glucose uptake and lactate product levels in OS cells. (k) Detection of protein levels of HK2 and PKM2 in OS cells by western blotting. *P < 0.05.

Subsequently, the biological function of circATP2A2 in OS was further surveyed. The interference efficiency of sh-circATP2A2 in OS cells is presented in Figure 2a. Downregulation of circATP2A2 decreased the OS cell growth and colony formation in MTT and plate clone assays (Figure 2b–d). circATP2A2F inhibition induced cell cycle arrest in OS cells in flow cytometry assay (Figure 2e and f). The migration and invasion of OS cells were also remarkably repressed in transwell assays (Figure 2g and h). In addition, the levels of glucose uptake and lactate product were lower in OS cells with low circATP2A2 expression (Figure 2i and j). HK2 and PKM2 are the key rate-limiting enzymes for glycolysis [23]. HK2 catalyzes the conversion of glucose to 6-phosphate glucose (G-6-P), while PKM2 catalyzes phosphoenolpyruvate (PEP) to produce ATP and pyruvate [24]. As expected, the levels of HK2 and PKM2 protein were downregulated in circATP2A2-repressed OS cells (Figure 2k). The results indicated that circATP2A2 promoted OS cell malignancy and glycolysis.

### 3.3 circATP2A2 acted as a miR-335-5p sponge

**Figure 3:**
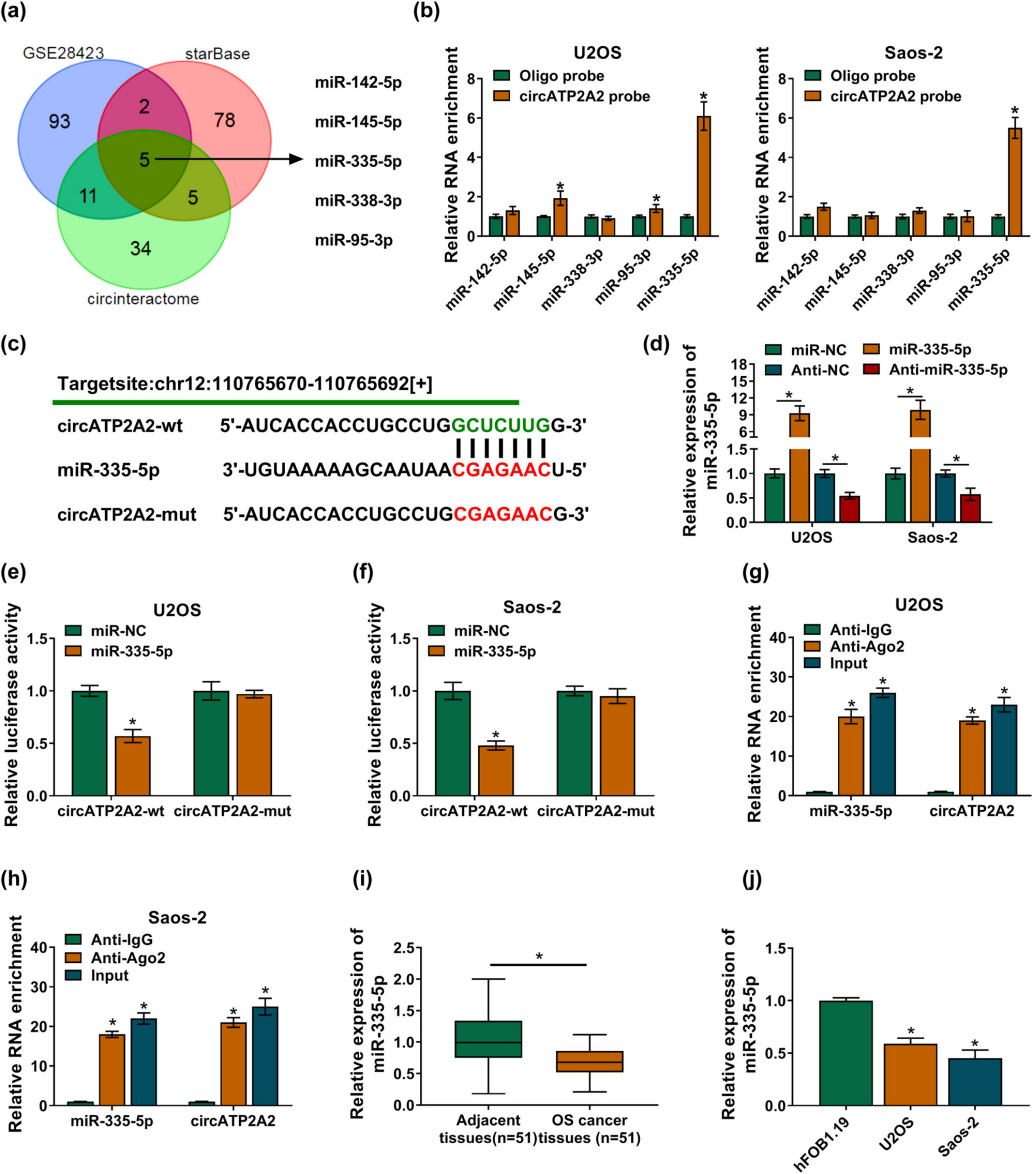
circATP2A2 was verified as a miR-335-5p sponge. (a) The candidate miRs containing the putative binding sites of circATP2A2 were overlapped in circinteractome, starBase, and GSE28423 databases. (b) The abundance of candidate miRs in the oligo probe and circATP2A2 probe groups was detected by RT-qPCR. (c) The putative binding sites of circATP2A2 on the miR-335-5p seed sequence. (d) The overexpression and interference efficiencies of miR-335-5p and Anti-miR-335-5p were analyzed by RT-qPCR. (e and f) The luciferase activity of circATP2A2-wt or circATP2A2-mut reporter in OS cells was assessed by dual-luciferase reporter assay. (g and h) The enrichment of miR-335-5p and circATP2A2 in co-immunoprecipitated in the anti-Ago2 and anti-IgG groups was analyzed by RT-qPCR. (i and j) The expression of miR-335-5p in OS tissues and cells was detected by RT-qPCR. *P < 0.05.

Through bioinformatics analysis, we discovered that circATP2A2 might be a sponge for 5 miRs (Figure 3a). Also, miR-335-5p could be pulled down by the circATP2A2 probe (Figure 3b). We then constructed luciferase reporter vectors containing circATP2A2-wt or circATP2A2-mut (Figure 3c). The overexpression and interference efficiencies of miR-335-5p and anti-miR-335-5p were verified by RT-qPCR (Figure 3d). The luciferase activity of the luciferase reporter vectors containing circATP2A2-wt was repressed in miR-335-5p-elevated OS cells (Figure 3e and f). Furthermore, circATP2A2 and miR-335-5p were co-immunoprecipitated in the anti-Ago2 group (Figure 3g and h). Additionally, miR-335-5p expression was evidently reduced in OS tissues and cells (Figure 3i and j). Collectively, these findings indicated that circATP2A2 acted as a decoy for miR-335-5p.

### 3.4 circATP2A2 adsorbed miR-335-5p to accelerate OS cell malignancy and glycolysis

**Figure 4:**
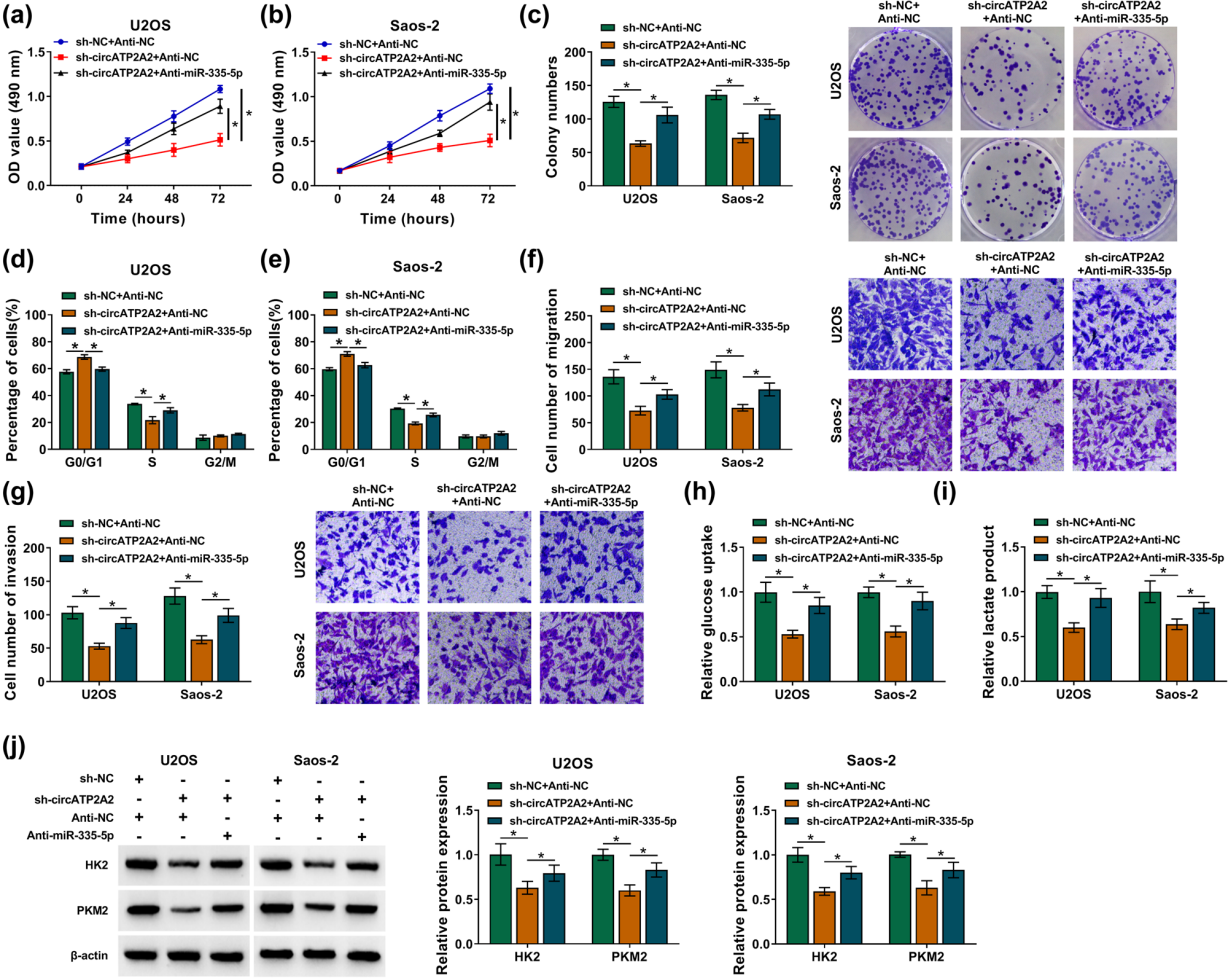
circATP2A2 regulated OS cell malignancy and glycolysis by sponging miR-335-5p. (a–j) OS cells were transfected with sh-NC + Anti-NC, sh-circATP2A2 + Anti-NC, or sh-circATP2A2 + anti-miR-335-5p. (a–g) The proliferation, cell cycle progression, migration, and invasion of OS cells were determined by MTT, plate clone, flow cytometry, and transwell assays, respectively. (h and i) Analysis of glucose uptake and lactate product levels in OS cells. (j) Protein levels of HK2 and PKM2 in OS cells were measured by Western blotting. *P < 0.05.

To validate whether circATP2A2 regulated OS cell malignancy and glycolysis via serving as a miR-335-5p decoy, we co-transfected sh-circATP2A2 and anti-miR-335-5p into OS cells. The results exhibited that miR-335-5p silencing restored the suppressive impact of circATP2A2 knockdown on growth, colony formation, and cell cycle progression of OS cells (Figure 4a–e). Moreover, the miR-335-5p inhibitor abrogated the inhibition of migration and invasion of OS cells induced by circATP2A2 silencing (Figure 4f and g). Furthermore, the decreases of glucose uptake and lactate product in circATP2A2-suppressed OS cells were overturned after the anti-miR-335-5p introduction (Figure 4h and i). Furthermore, the downregulation of HK2 and PKM2 in OS cells with low circATP2A2 expression was restored by miR-335-5p silencing (Figure 4j). Therefore, these data disclosed that circATP2A2 promoted OS cell malignancy and glycolysis by adsorbing miR-335-5p.

### 3.5 MYH9 was a target of miR-335-5p

**Figure 5:**
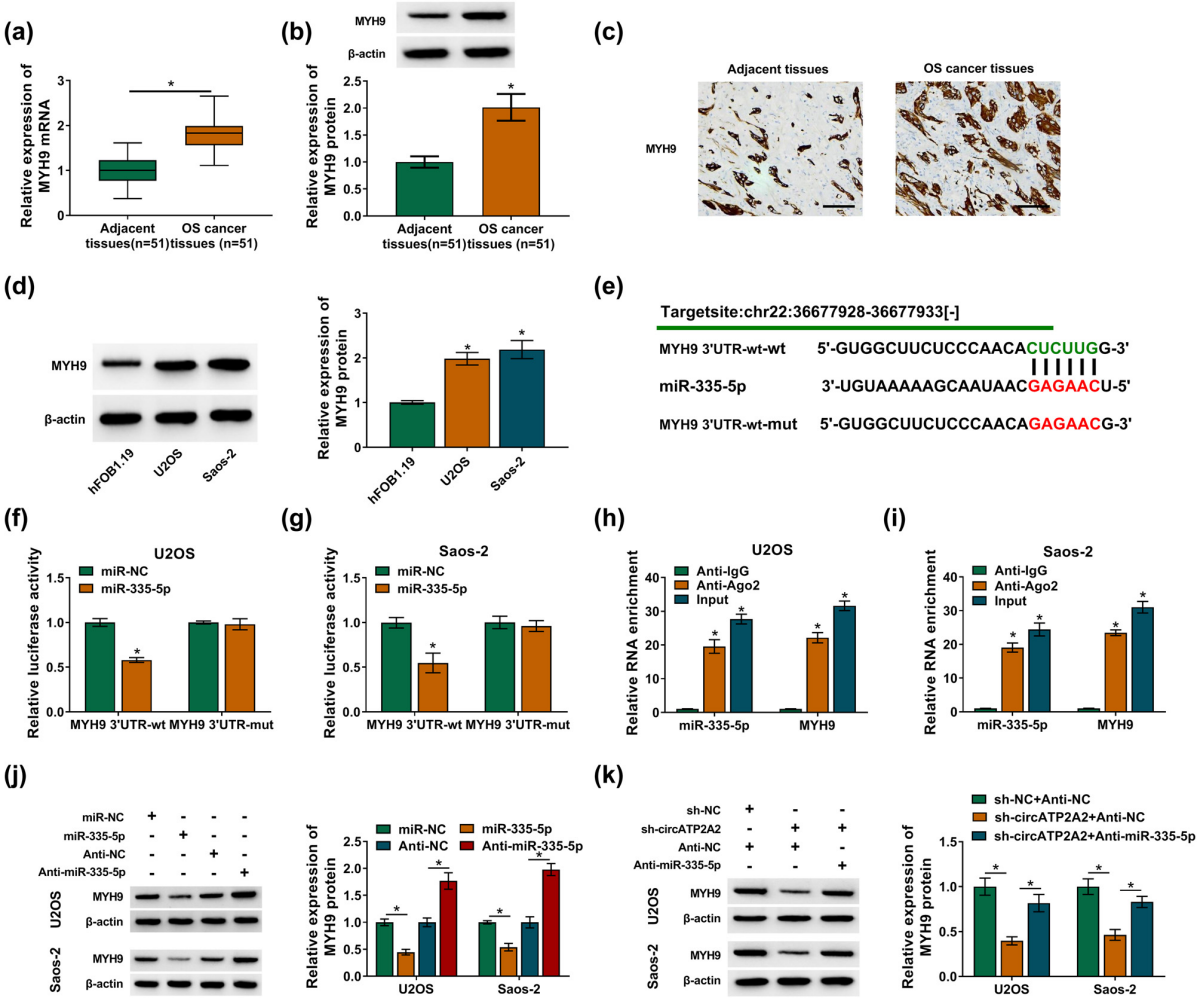
MiR-335-5p targeted MYH9. (a–c) The levels of MYH9 mRNA and protein in OS tissues were analyzed by RT-qPCR, western blotting, and IHC. (d) The level of MYH9 protein in OS cells was analyzed by western blotting. (e) The binding sites between MYH9 and miR-335-5p were predicted by the starBase database. (f and g) The luciferase activities of the reporter plasmids carrying MYH9 3′UTR-wt and MYH9 3′UTR-mut were determined with dual-luciferase reporter assay. (h and i) The abundance of miR-335-5p and MYH9 in the immunoprecipitates of the anti-Ago2 and anti-IgG groups was assessed by RT-qPCR. (j) The impacts of miR-335-5p overexpression and silencing on the level of MYH9 protein were analyzed by Western blotting. (k) The level of MYH9 protein in OS cells transfected with sh-NC + anti-NC, sh-circATP2A2 + anti-NC, or sh-circATP2A2 + anti-miR-335-5p was detected by Western blotting. *P < 0.05.

Previous research studies revealed that MYH9 acted as an oncogene in OS. To survey the relationship between MYH9 and miR-335-5p, we first verified MYH9 expression in OS tissues. The results presented that the levels of MYH9 mRNA and protein were increased in OS tissues (Figure 5a–c). As expected, the level of MYH9 protein was also elevated in OS cells (Figure 5d). Through bioinformatics prediction, we discovered that MYH9 might be a target for miR-335-5p (Figure 5e). Furthermore, the luciferase activity of the MYH9 3′UTR-wt reporter was remarkably decreased in OS cells with overexpression of miR-335-5p (Figures 5f and g). Also, the abundance of miR-335-5p and MYH9 in the immunoprecipitates of the anti-Ago2 group was significantly increased (Figure 5h and i). In addition, miR-335-5p overexpression repressed the protein level of MYH9 in OS cells, while miR-335-5p inhibition had an opposing impact (Figure 5j). There was also downregulation of MYH9 protein in OS cells with the inhibition of circATP2A2, whereas this downregulation was restored after miR-335-5p silencing (Figure 5k). Therefore, circATP2A2 could modulate MYH9 expression by sponging miR-335-5p.

### 3.6 MiR-335-5p repressed OS cell malignancy and glycolysis by targeting MYH9

**Figure 6:**
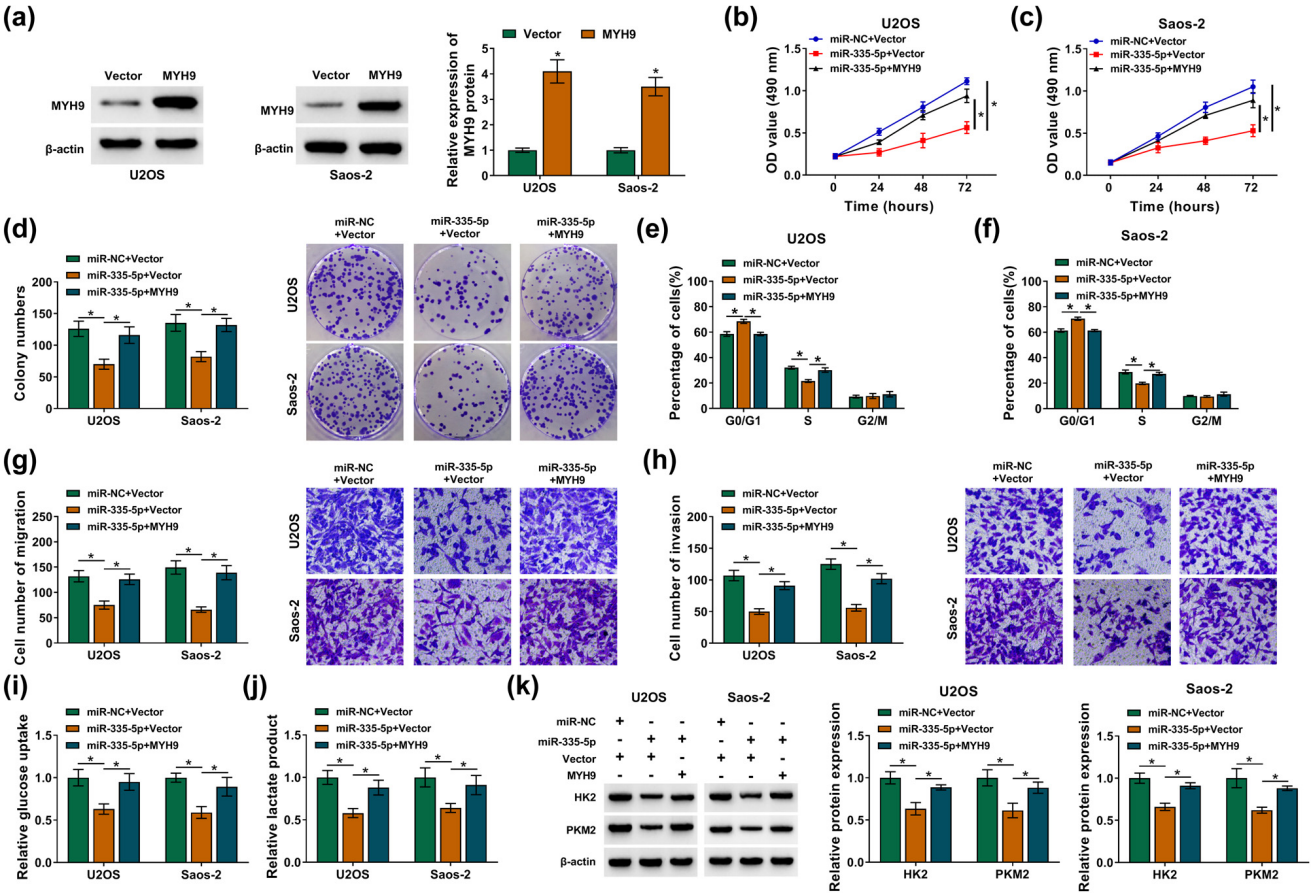
MiR-335-5p targeted MYH9 to inhibit OS cell malignancy and glycolysis. (a) The overexpression efficiency of MYH9 in OS cells was detected by Western blotting. (b–k) OS cells were transfected with miR-NC + vector, miR-335-5p + vector, or miR-335-5p + MYH9. (b–h) The proliferation, cell cycle progression, migration, and invasion of OS cells were evaluated by MTT, plate clone, flow cytometry, and transwell assays, respectively. (i and j) Detection of glucose uptake and lactate product levels in OS cells. (k) Analysis of HK2 and PKM2 protein levels in U2OS and Saos-2 cells by Western blotting. *P < 0.05.

To investigate whether miR-335-5p affected OS cell malignancy and glycolysis via MYH9, we performed rescue experiments. As exhibited in Figure 6a, the MYH9 protein level was increased in OS cells after MYH9 transfection. Furthermore, miR-335-5p elevation constrained growth, colony formation, and cell cycle progression of OS cells but this repression was reversed after MYH9 overexpression (Figure 6b–f). Moreover, MYH9 upregulation reversed the repression of cell migration and invasion in OS cells induced by miR-335-5p overexpression (Figure 6g and h). Also, the elevation of MYH9 overturned the decrease of glucose uptake and lactate production in miR-335-5p-elevated OS cells (Figure 6i and j). In addition, the downregulation of HK2 and PKM2 in miR-335-5p-increased OS cells was restored after MYH9 upregulation (Figure 6k). These results proved that miR-335-5p curbed OS cell malignancy and glycolysis via targeting MYH9.

### 3.7 circATP2A2 knockdown repressed OS cell proliferation and glycolysis *in vivo*

**Figure 7:**
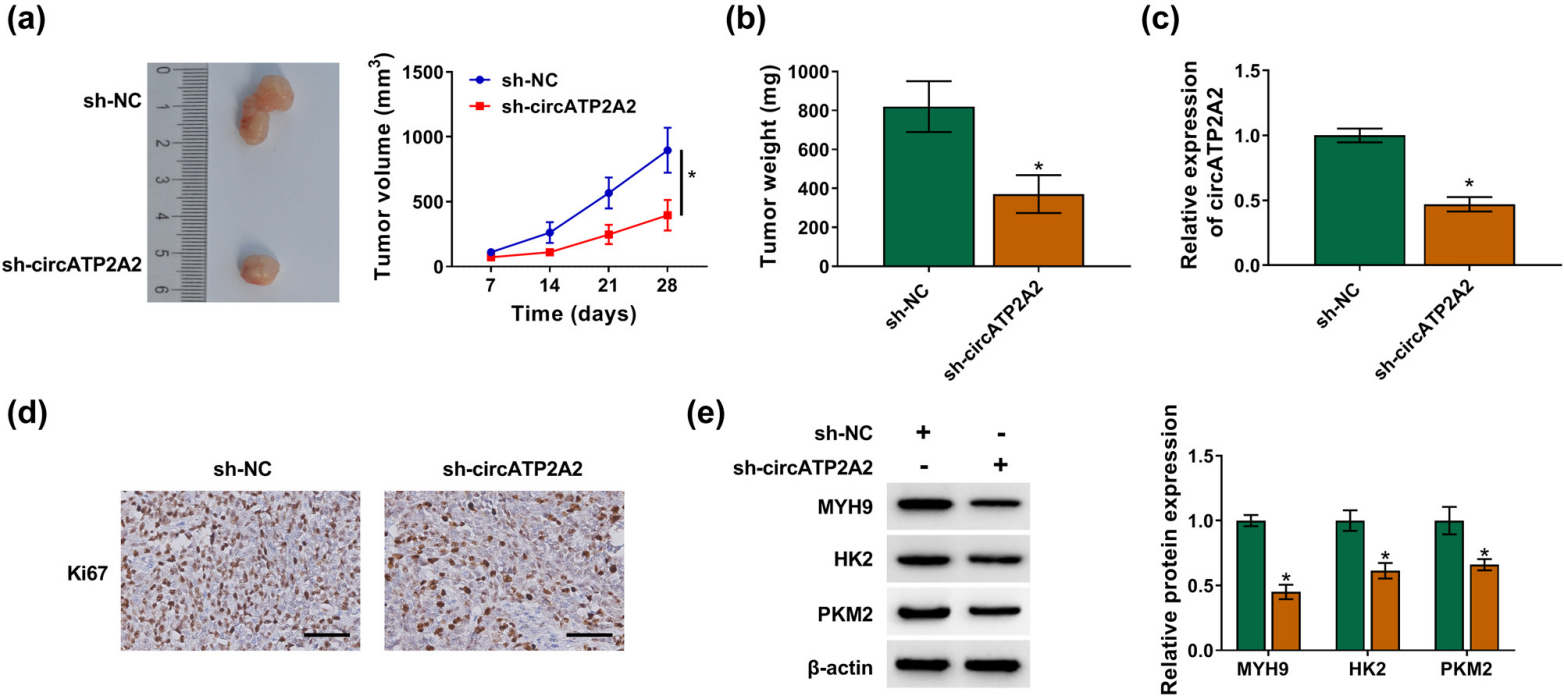
circATP2A2 inhibition decreased OS cell proliferation and glycolysis *in vivo*. (a and b) Tumor volume and weight of mice injected with OS cells carrying sh-circATP2A2 or sh-NC. (c) circATP2A2 expression in xenograft tumors in the sh-circATP2A2 and sh-NC groups was analyzed by RT-qPCR. (d) The level of Ki67 in xenograft tumors in the sh-circATP2A2 and sh-NC groups was analyzed by IHC. (e) Protein levels of MYH9, HK2 and PKM2 in xenograft tumors in the sh-circATP2A2 and sh-NC groups were detected by Western blotting. *P < 0.05.

The biological function of circATP2A2 in OS was further verified through xenograft assay. The results displayed that the volume and weight of xenograft tumors in mice injected with OS cells carrying sh-circATP2A2 were smaller and lower (Figure 7a and b). Also, circATP2A2 expression was decreased in xenograft tumors in mice injected with OS cells carrying sh-circATP2A2 (Figure 7c). IHC revealed that the expression of Ki67 was also reduced in xenograft tumors in the sh-circATP2A2 group (Figure 7d). In addition, the protein levels of MYH9, HK2, and PKM2 were downregulated in xenograft tumors in the sh-circATP2A2 group (Figure 7e). These data suggested that circATP2A2 silencing constrained OS cell proliferation and glycolysis *in vivo*.

## 4 Discussion

A series of studies have proved that circRNAs are promising diagnostic and prognostic biomarkers and therapeutic A targets for diverse tumors [25,26,27]. Our results exhibited that circATP2A2 was overexpressed in OS tissues and cells, which was in line with the report of Chen et al. [28]. Also, OS patients with high circATP2A2 expression displayed a worse overall survival, indicating that circATP2A2 might be a prognostic biomarker. Also, the AUC of circATP2A2 was 0.77, manifesting that circATP2A2 might be a diagnostic biomarker. In addition, circATP2A2 silencing repressed OS cell proliferation, migration, and invasion *in vitro*. Furthermore, circATP2A2 knockdown decreased the xenograft tumor growth *in vivo*, and the protein level of Ki67 (a good marker of proliferation marker) was lower in circATP2A2-knockdown xenograft tumors. These results suggested that circATP2A2 silencing decreased OS growth *in vivo*. Therefore, we inferred that circATP2A2 acted as a promoter in OS and might be a biomarker and target for OS diagnosis and treatment.

A characteristic of cancer cells is that their glucose metabolism changes from mainly oxidative respiration to aerobic glycolysis [29]. The elevation of glycolysis accelerates cancer cell angiogenesis and metastasis [30]. HK2 and PKM2, the rate-limiting enzymes in glycolysis, are involved in the conversion of glucose to pyruvate [24]. Herein, circATP2A2 inhibition reduced HK2 and PKM2 protein levels in OS cells *in vitro* and xenograft tumors *in vivo*. These data manifested that circATP2A2 promoted OS cell glycolysis.

Based on the ceRNA hypothesis that circRNAs can adsorb miRs by MREs, thus regulating the targets of miRs [31], we uncovered circATP2A2 as a miR-335-5p decoy. Wang et al. reported that DANCR adsorbed miR-1972 and miR-335-5p to elevate ROCK1 expression, resulting in facilitating OS cell growth and metastasis [32]. Also, TUG1 elevated ROCK1 expression by serving as a miR-335-5p decoy, thereby accelerating OS cell invasion and migration [33]. These results illustrated that miR-335-5p played an anti-tumor tumor activity in OS. Our data also presented that miR-335-5p silencing reversed the suppressive impacts of circATP2A2 silencing on OS cell malignancy and glycolysis. Thus, we concluded that circATP2A2 acted as a miR-335-5p decoy and promoted OS cell malignancy and glycolysis by sequestering miR-335-5p.

In addition, MYH9 was validated as a miR-335-5p target. Wang et al. discovered that MYH9 could activate the MAPK/AKT pathway, resulting in colorectal cancer cell growth and metastasis [34]. Also, the negative feedback loop of miR-6089/MYH9/β-catenin/c-Jun constrained the growth of ovarian cancer [35]. Zhou et al. showed that MYH9 expression was strikingly increased in OS, and high MYH9 expression was associated with OS cell invasion and metastasis [36]. Also, MRPL23-AS1 accelerated carcinogenesis and tumor growth via increasing MYH9 by sponging miR-30b in OS [37]. In our study, MYH9 elevation reversed the inhibitory impacts of the miR-335-5p mimic on OS cell malignancy and glycolysis. Additionally, MYH9 was regulated by the circATP2A2/miR-335-5p axis. Therefore, we concluded that circATP2A2 promoted OS cell malignancy and glycolysis through upregulating MYH9 expression by sponging miR-335-5p (Graphical abstract image).

## 5 Conclusion

circATP2A2 played a carcinogenic role in OS. Furthermore, circATP2A2 promoted malignant behaviors and glycolysis by serving as a miR-335-5p decoy and elevating MYH9 expression. This research provided a new mechanism of circATP2A2 responsible for tumor growth and glycolysis in OS.
